# Mitochondrial DNA Content Varies with Pathological Characteristics of Breast Cancer

**DOI:** 10.1155/2011/496189

**Published:** 2011-10-17

**Authors:** Ren-Kui Bai, Julia Chang, Kun-Tu Yeh, Mary Ann Lou, Jyh-Feng Lu, Duan-Jun Tan, Hao Liu, Lee-Jun C. Wong

**Affiliations:** ^1^Department of Molecular and Human Genetics, Baylor College of Medicine, One Baylor Plaza, NAB 2015, Houston, TX 77030, USA; ^2^GeneDx, Inc., 207 Perry Parkway, Gaithersburg, MD 20877, USA; ^3^Department of Pathology, Changhua Christian Hospital, ChangHua, Taiwan; ^4^Department of Surgery, Cardinal Tien Hospital, Taipei, Taiwan; ^5^School of Medicine, Fu Jen Catholic University, Tio, Taipei, Taiwan; ^6^Department of Pathology, SUNY Downstate Medical Center, Brooklyn, NY 11203, USA; ^7^Division of Biostatistics, Dan L. Duncan Cancer Center, Baylor College of Medicine, Houston, TX 77030, USA; ^8^Cancer Prevention and Population Science Program, Dan L. Duncan Cancer Center, Baylor College of Medicine, Houston, TX 77030, USA

## Abstract

Changes in mitochondrial DNA (mtDNA) content in cancers have been reported with controversial results, probably due to small sample size and variable pathological conditions. In this study, mtDNA content in 302 breast tumor/surrounding normal tissue pairs were evaluated and correlated with the clinico-pathological characteristics of tumors. Overall, mtDNA content in tumor tissues is significantly lower than that in the surrounding normal tissues, *P* < 0.00001. MtDNA content in tumor tissues decreased with increasing tumor size. However, when the tumor is very large (>50 cm^3^), mtDNA content started to increase. Similarly, mtDNA content decreased from grades 0 and I to grade II tumors, but increased from grade II to grade III tumors. Tumors with somatic mtDNA alterations in coding region have significantly higher mtDNA content than tumors without somatic mtDNA alterations (*P* < 0.001). Tumors with somatic mtDNA alterations in the D-Loop region have significantly lower mtDNA content (*P* < 0.001). Patients with both low and high mtDNA content in tumor tissue have significantly higher hazard of death than patients with median levels of mtDNA content. mtDNA content in tumor tissues change with tumor size, grade, and ER/PR status; significant deviation from the median level of mtDNA content is associated with poor survival.

## 1. Introduction

Mitochondria are the small power houses of the human cells. They consume oxygen to generate about 80%~90% of the energy supply for the cell in the form of ATP and much of the endogenous reactive oxygen (ROS) via oxidative phosphorylation (OXPHOS). Mitochondria also play an important role in programmed cell death through the release of cytochrome c from mitochondria to trigger a proteolytic cascade involving caspases. Mitochondria have their own genome. Depending on the cell type, each cell contains hundreds to thousands copies of mitochondrial DNA (mtDNA). The mtDNA content is related to the energy demand of each cell type. For example, each muscle cell contains several thousands of copies of mtDNA molecules, while each skin cell contains several hundreds of copies. Human mtDNA encodes 13 protein subunits of the respiratory chain as well as 2 rRNAs and a set of 22 tRNAs, essential for mitochondrial protein synthesis [1].

About eighty years ago, Warburg observed an increased capacity of aerobic glycolysis in cancers, it has been postulated that cancer might be the result of altered mitochondrial function [2, 3]. Subsequent studies revealed that the mitochondrial functions (OXPHOS) are downregulated in many cancers [4]. Recently, alterations in mtDNA content have been reported in a variety of cancers, but the results are controversial. Kim et al. [5] reported that mitochondrial DNA quantity increases with histopathological grade in premalignant and malignant head and neck lesions. Using quantitative real-time PCR analysis to study mtDNA contents in 59 cases of paired invasive tumor and surrounding nontumor breast tissues, Yu et al. showed that reduced mitochondrial DNA copy number was correlated with tumor progression and prognosis in Chinese breast cancer patients [6]. In human hepatocellular carcinoma (HCC), the mtDNA content was found to be significantly reduced in female HCC but not in male HCC [7]. Studies of primary epithelial ovarian carcinomas revealed that mtDNA contents in tumor cells were significantly higher than those in normal ovarian tissue. The average mtDNA content in pathologically low-grade tumors was over twofold higher than that in high-grade carcinomas [8]. These authors concluded that there is an association of decreased mtDNA with ovarian cancer progression [8]. mtDNA depletion was also reported to be associated with tumor aggressiveness in renal cell carcinoma [9] and with less-differentiated gastric carcinoma [10]. The explanations for these controversial results could be tissue specificity or stage- and class-specific changes of mtDNA copy number [11]. It is also possible that the discrepancy is due to small sample size of these studies. 

In this report, we investigated the mtDNA content in 302 breast tumors and their corresponding surrounding pathologically normal tissues. We also correlated the mtDNA contents with the clinicopathological features.

## 2. Materials and Methods

### 2.1. DNA Samples

Total DNA was isolated from 302 breast tumors and their surrounding normal tissues using proteinase K digestion, phenol-chloroform extraction, ethanol precipitation method. The surrounding normal tissue was defined as pathologically and morphologically normal, without evidence of hyperplasia. Alterations at molecular level were not examined other than the studies presented in this report. This study was carried out according to Institutional Review Board (IRB) approved protocols. Informed consent was obtained from each patient whose samples were included in the study when the samples were collected.

### 2.2. Determination of mtDNA Content by Real-Time Quantitative PCR

The real-time PCR reaction was performed in duplicate for each mitochondrial and nuclear probe using primers, mtF3212 and mtR3319 for mitochondrial tRNA-Leu (UUR) gene and F589 and R674 for beta 2 microglobulin nuclear gene (B2M) as previously described [12]. The copy number ratio of mtDNA to B2M gene (mtDNA/B2M) calculated using delta Ct method was used as mtDNA content [12]. The tumor/normal ratio (T/N ratio) of mtDNA content indicates the ratio of the mtDNA content in tumor tissue to the mtDNA content in the surrounding normal tissue from the same patient. T/N ratio > 1 means the mtDNA content increases in tumor tissue compared to the surrounding normal tissue. T/N ratio <  1 means the mtDNA content decreases in the tumor tissue compared to the surrounding normal tissue.

### 2.3. Screening of Somatic mtDNA Changes in Tumors

For a subset of 74 pairs of samples, somatic mtDNA alterations in tumors were screened by temporal temperature gradient gel electrophoresis (TTGE) as previously described [13–16]. The subset of samples analyzed for mtDNA alterations were chosen randomly from and is similar in clinico-pathological features (similar proportion of patients have the same features in terms of the age group, tumor type, tumor size group, pathological grade, TNM stage; Fisher's exact *P* values are all greater than 0.5) to the entire sample set, so that the subset of samples may represent the entire sample set. The PCR products of DNA fragments from tumor and its surrounding normal tissues were analyzed side by side. On TTGE analysis, a single band shift represents a homoplasmic DNA alteration, and a multiple-banding pattern represents a heteroplasmic change. 

### 2.4. Identification of Somatic mtDNA Alterations by DNA Sequencing

DNA fragments showing differences in TTGE banding patterns between tumors and the surrounding normal tissues were sequenced to identify the exact changes [13–16]. The mtDNA sequence differences between the tumor and the surrounding normal tissue were scored as somatic alterations. 

### 2.5. Statistical Analysis

The mtDNA content or T/N ratio among different groups within a given variable (e.g., age, size, and grade) was compared using nonparametric tests (Mann-Whitney test or Kruskal-Wallis test). Fisher's exact test was used to correlate the change (increase or decrease) in mtDNA content with the presence of somatic alterations in coding or noncoding D-loop regions. Cox proportional-hazards survival regression analysis was used to investigate the relationship between the survival time, clinicopathological variables, mtDNA content, and T/N ratio of mtDNA content using both univariate and multivariate models. The outcome was the overall survival, defined from date of tumor diagnosis to the death of any cause. Hazard ratios were presented with their 95% confidence intervals (95% CI). All statistical tests were two sided. *P* values less than 0.05 are considered statistically significant. All the tests were performed using the SPSS 13.0 (SPSS Inc. Chicago, Illinois). 

## 3. Results

### 3.1. Reduced mtDNA Content in the Majority of Tumor Tissues

In a total of 302 tissue pairs, 9 pairs were excluded from statistical analysis due to real-time PCR failure for DNA samples from either the tumor tissue or the surrounding normal tissue. In the remaining 293 pairs, the mtDNA content in tumor tissues (583 ± 511) is significantly lower than that in the surrounding normal tissues (688 ± 389), *P* = 3.069e − 6. About 65% (191/293) of tumor tissues have reduced mtDNA content when compared to their surrounding normal tissue (T/N ratio < 1). About 53% (155/293 = 52.9%) of tumor tissues, the mtDNA content decrease to lower than 80% of that in the surrounding normal tissue counterpart, while only around 25% (74/293) of tumor tissues have T/N ratios > 1.2. The mtDNA content in tumor tissue and the T/N ratio does not correlate with the age of the patient (Table 1).

### 3.2. The mtDNA Content in Tumor Tissue Varies with Tumor Size

**Figure 1:**
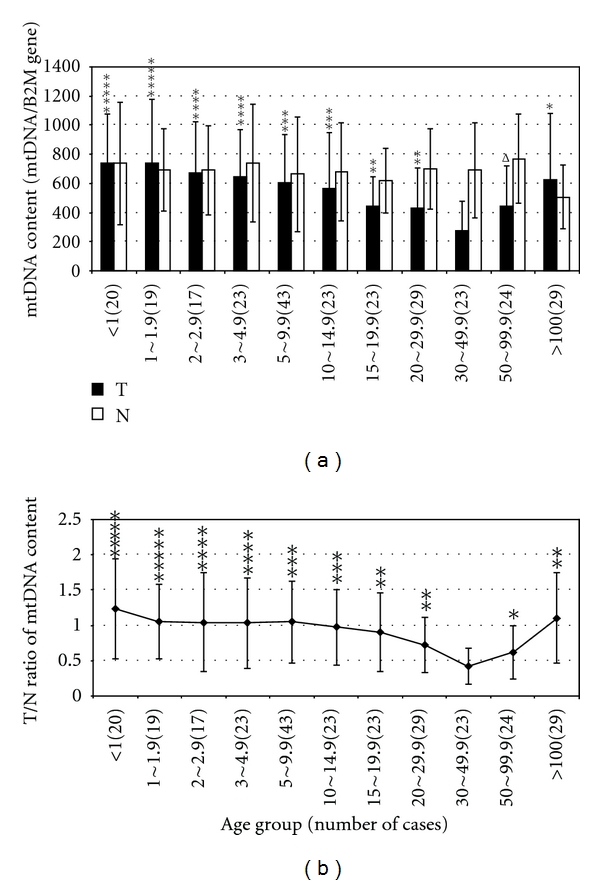
Comparison of the mtDNA content and the T/N ratio in tumors of different size. (a) mtDNA content of tumor tissues and their surrounding pathologically normal tissues, in tumors of different size. (b) T/N ratio of tumors with different size. T: mtDNA content of the tumor tissue; N: mtDNA content of the pathologically normal tissue surrounding the tumor tissue; T/N ratio: the ratio of mtDNA content of the tumor tissue to that of the pathologically normal tissue surrounding the tumors from the same individual; *X*-axis represents tumor size in cubic centimeter (totally 11 groups); the *Y*-axis represents mtDNA content (mtDNA/B2M gene) and T/N ratio respectively in (a) and (b); the results from other size groups were compared with those from the group of tumors sized 30–49.9 cubic centimeters; the symbols of, ^Δ^, *, **, ***, **** and ***** represent significant *P* values of <0.1, <0.05, <0.01, <0.001, <0.0001 and <0.00001, respectively.

When mtDNA content was plotted against tumor size, it was noticed that mtDNA content in tumor tissue decreased with increased tumor size up to 50 cm^3^ (Figure 1). However, when the tumor size is >50 cm^3^, the mtDNA content in tumor started to increase (Figure 1). The average mtDNA content in tumors sized 30–49.9 cm^3^ was significantly lower when compared to mtDNA content in smaller or larger tumors (Figure 1 and Table 1). In contrast, the mtDNA content in the surrounding normal tissue remained relatively constant regardless of tumor size (Figure 1). Similar to mtDNA content in tumor, the T/N ratio also varied with the tumor size (Figure 1 and Table 1). 

### 3.3. Correlation of mtDNA Content with the Pathological Grade and Stage of Tumors

**Figure 2:**
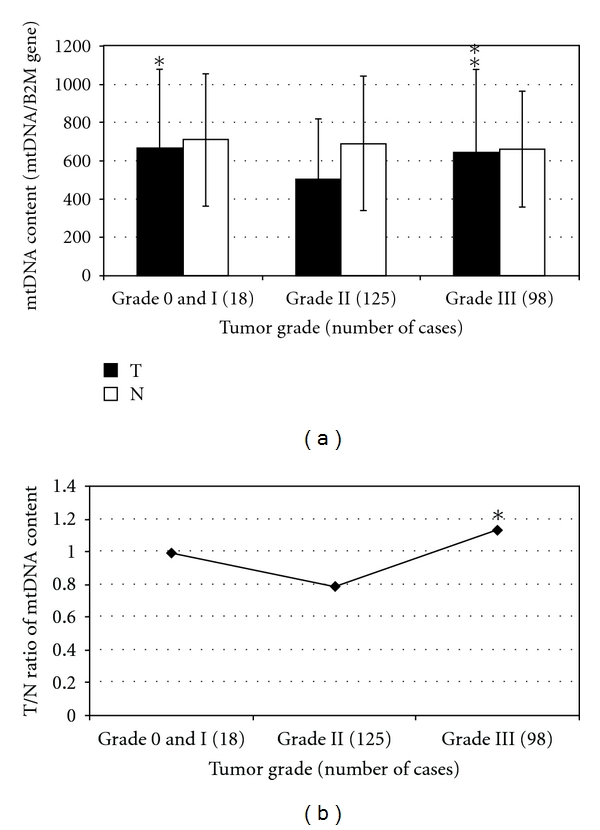
Comparison of the mtDNA content and the T/N ratio in tumors of different grade. (a) mtDNA content of tumor tissues and their surrounding pathologically normal tissues, in tumors of different grade. (b) T/N ratio of tumors with different grade. T, mtDNA content of the tumor tissue; N: mtDNA content of the pathologically normal tissue surrounding the tumor tissue; T/N ratio: the ratio of mtDNA content of the tumor tissue to that of the pathologically normal tissue surrounding the tumors from the same individual; *X*-axis represents tumor histological grade; the *Y*-axis represents mtDNA content (mtDNA/B2M gene) and T/N ratio respectively, in (a) and (b); the results from tumors of grades 0 and I and grade III were compared with those from tumors of grade II; The symbols * and ** represent significance *P* value of <0.05 and <0.01, respectively.

The mtDNA contents in tumor tissues and the T/N ratios were significantly higher in grade III tumors than in grade II tumors, *P* = 0.006 (Table 1 and Figure 2). There were fewers grades 0 and I tumors (18/241 = 7.5%). The mtDNA content in grade 0-I tumors was also significantly higher when compared to grade II tumors (*P* = 0.019, Table 1 and Figure 2). The mtDNA content does not correlate with tumor stage. However, the mtDNA content in tumors at stages 0-I or stage II is lower than that in tumors at stage III~IV, *P* = 0.073. 

### 3.4. mtDNA Content and Expression Level of ER, PR, and HER2 

In the presence of ER, the mtDNA content in tumors is higher than mtDNA content in ER-negative tumors, but it does not reach the statistical significant level (*P* = 0.103). However, the T/N ratio is significantly lower in ER negative tumors than in ER-positive tumors (*P* = 0.047) (Table 1). Likewise, both the mtDNA content and the T/N ratio are borderline significantly lower in the PR-negative tumors than in PR-positive tumors (*P* = 0.061 and 0.059, resp.) (Table 1). Similarly, for HER2, the T/N ratio is significantly lower in HER2-negative tumors than in HER2-positive tumors (*P* = 0.023) (Table 1). The mtDNA content in ER- and PR-positive (ER+/PR+) tumors is also significantly higher than in ER/PR both negative or either positive tumors (*P* = 0.005, Table 1). 

### 3.5. Somatic mtDNA Alterations

Of the 74 tumor/normal tissue pairs analyzed, somatic alterations were detected in 34 tumors (34/74 = 46%); 19 had somatic alterations in the non-coding D-loop region only, 15 harbored alterations in coding region. Seven of the 15 with coding region changes also contained alterations in D-loop region. The detail of somatic mtDNA changes are listed in Table S1 of the supplementary material available online at doi:10.1155/2011/496189.

### 3.6. mDNA Content and Somatic mtDNA Alterations

As shown in Table 2, the mtDNA content and the T/N ratio in tumors with somatic mtDNA alterations in coding regions, are significantly higher than in tumors without somatic mtDNA alterations (*P* < 0.001) or with alterations in the D-loop region (*P* < 0.001). The proportion of tumors with T/N ratio greater than 1.2 is also significantly higher in tumors with somatic mtDNA alterations in coding regions than in tumors without somatic mtDNA alterations (*P* = 0.033) or with alterations in the non-coding D-loop region (*P* = 0.001). In contrast, the mtDNA content and the T/N ratio in tumors with somatic mtDNA alterations in the non-coding D-loop region, are significantly lower than those in tumors without somatic mtDNA alterations, *P* < 0.001. Meanwhile, the proportion of tumors with T/N ratio greater than 1.2 is lower in tumors with somatic mtDNA alterations in the non-coding D-loop region than in tumors without somatic mtDNA alterations (*P* = 0.081). These results suggest that mtDNA alterations in coding regions, but not in the non-coding D-loop region, tend to induce compensatory mtDNA biogenesis. The majority of the tumors without somatic mtDNA alterations had reduced mtDNA content compared to surrounding normal tissue. 

### 3.7. Linking mtDNA Content to Clinicopathological Features and Patients' Survival

In univariate Cox regression analysis, patients with tumors of higher histological grade, or larger tumor size, or in late TNM stage had a significantly shorter survival (higher hazard of death) rate; *P* values are 0.014, 0.004, and 0.011, respectively (Table 3). This is consistent with the previous report that high histopathological grade, large tumor size, and late TNM stage are all prognostic factors for survival [17]. Patients with low mtDNA content in tumor had significantly lower hazard of death than patients with median (relatively normal) level of mtDNA content in tumor (*P* = 0.042). After adjusting for the factors including tumor grade, tumor size, and patients' age, the hazard ratio of patients with both low and high mtDNA content in the tumor tissue became significantly higher compared to patients with median level of mtDNA content in the tumor tissue (HR = 3.39, 95% CI = 1.17–9.82, *P* = 0.025 for patients with low mtDNA content; HR = 4.05, 95% CI = 1.27–12.87, *P* = 0.018 for patients with high mtDNA content in the tumor tissue) (Table 3). There is no correlation between the hazard ratio and T/N ratio.

## 4. Discussion

Mitochondria play a central role in cellular energy production. Approximately 90% of cellular energy is produced by the electron transport chain embedded in the inner mitochondrial membrane though the process of oxidative phosphorylation [18–20]. Although many factors, including the mutual interaction between mitochondrial energy state, biogenesis, metabolite, dynamics, and degradation are involved in the regulation of cellular and mitochondrial energy production [21], the interplay between nuclear and mitochondrial genes may hold the final understanding of the mitochondrial role in the disease processes [22]. Altered mitochondrial function in cancer cells has been recognized and postulated to be the fundamental cause of cancer [2, 3]. Downregulation of mitochondrial oxidative phosphorylation is a common phenotype in cancers [4]. However, only a few reports link altered mitochondrial function directly to deleterious mtDNA point mutations [23–25].

Altered mitochondrial function can be caused by qualitative or quantitative changes in nuclear or mitochondrial encoded genes. Primary mitochondrial DNA depletion syndrome is a group of severe autosomal recessive, heterogeneous clinical syndromes caused by mutations in nuclear-encoded proteins responsible for mitochondrial DNA biosynthesis or the maintenance of mitochondrial deoxynucleotide pools [26–30]. Cancer has not been the main clinical feature of mtDNA depletion syndrome. However, our recent study [27] revealed that infant patients with mutations in *DGUOK* (deoxyguanosine kinase) *MPV17* genes had developed hepatocellular carcinoma (HCC) [26–28, 30]. Thus, it is possible that mtDNA depletion is associated with HCC in young age. Most patients with congenital mtDNA depletion syndrome died at young age (<2 years) before cancer developed. 

Reduction of mtDNA in tumor tissues have been reported in a variety of human cancers, including HCC [7, 31, 32], breast [6, 33], gastric [10], and other cancers [14, 34]. However, contradictory observations have also been reported [35, 36]. Depletion of mtDNA such as rho (0) cells showed diminished proliferation and tumorigenicity [35, 36]. Although mitochondrial membrane potential (Ψm) was markedly reduced, the apoptotic rate of T47D rho(0) cells remained the same as that of their parental cells [36]. Clinically, overall increased mtDNA content had been reported in premalignant and malignant head and neck lesions [5] and ovarian cancer [37]. For this reported overall decrease of mtDNA content in tumors, there was always increased mtDNA content in some of the samples studied [6, 7, 10, 14, 31–34]. Most of these studies had sample size less than 100. 

In this report, we studied the mtDNA content in 302 pairs of tumor/normal breast tissue. Overall, the mtDNA content in tumor tissues was significantly lower than the mtDNA content in the surrounding normal tissues. About 53% of tumor tissues have reduced mtDNA content (T/N ratio < 0.8) compared to surrounding normal tissue, while only about 25% tumors have higher mtDNA contents (T/N ratio > 1.2) than their surrounding normal tissues. This is consistent with most of the published studies [6, 7, 10, 14, 31–34] that reported that between 22.6% and 63% of tumor tissues had decreased mtDNA content. In addition, we studied mtDNA content in 20 pairs of tumor/normal tissues each of esophageal, liver, lung, and oral cancers (unpublished data). The results were consistent with what was observed in breast cancers in this study. The change, increase or decrease, in mtDNA content in tumors apparently depends on the stage of tumor progression, histopathological grades, size of tumors, and the expression of estrogen and progesterone receptors [5, 8, 33]. In contrast to the small size of the studies performed by other researchers, the large sample size of our study allows us to compare the mtDNA content in tumors of different clinico-pathological subgroups. Similar to our results, Yamada et al. also observed a decrease in mtDNA content with increasing tumor size in HCC [32]. The underlying mechanism for this decrease of mtDNA content with increased tumor size is not clear. Oxygen can initiate respiration and mitochondrial biogenesis [38]. Whereas under hypoxic condition in cancer cells, hypoxia inducible factor (HIF) inhibits mitochondrial biogenesis [39] or disrupts mitochondria by mitophagy [40]. When tumor is growing in size, cells are becoming more hypoxic, mitochondrial biogenesis is decreased. An alternative explanation for the decreased mtDNA content in tumor tissues with increased tumor size could be that unchanged mtDNA biosynthesis rate is unable to catch up with the accelerated cellular proliferation in tumors. However, when the tumor grows very large (>50 cm^3^) and becomes invasive, mtDNA content increases with tumor size (Figure 1), possibly because initially during the tumor size increase and the mtDNA content decrease, the function of the mitochondria is increasingly damaged. When the tumor becomes very large and invasive, the mitochondrial function is severely damaged either due to very low mtDNA content or mutations in the mtDNA or nuclear genes; the induction of HIF under hypoxia condition is inhibited [41] and mtDNA biogenesis is no longer inhibited, so the mtDNA content starts to increase. Increased mtDNA content in the extremely large tumors may also be a compensatory response to severely damaged mitochondrial function [42, 43]. Interestingly, breast tumors with increased mtDNA content have a lower sensitivity to chemotherapy [44].

Synthesis of mitochondrial DNA is fully controlled by nuclear encoded genes. These genes may be under the regulation of tumor suppressor or oncogenes. For example, the small subunit of p53-inducible ribonucleotide reductase, RRM2B, is a heterotetrameric enzyme responsible for the de novo synthesis of deoxyribonucleoside diphosphates that are essential for DNA synthesis during cell cycle checkpoint for DNA damage [45, 46]. Thus, cancer cells bearing *p53* or *RRM2B* mutations could theoretically affect mtDNA biosynthesis, leading to mtDNA depletion. Similarly, HCC was found in a patient with hepatocerebral form of mtDNA depletion syndrome caused by mutations in *DGUOK* gene responsible for mitochondrial salvage synthesis of deoxynucleotides [47]. These results suggest a possible link between mtDNA depletion and tumorigenesis.

Our results also showed that the mtDNA content was significantly higher in high grade (Grade III) breast tumors than those in lower grade (Grade II) tumors. It is possible that in high grade tumors HIF expression is inhibited and mitochondrial biogenesis and mtDNA biosynthesis is no longer inhibited [41]. Alternatively, expression of oncogenes, growth factors, and metastatic proteins, during tumor progression, may also regulate mitochondrial biogenesis. This is evident by a recent report [8] that shows that epithelial tumors could be categorized to type I and type II tumors. Mutations in KRAS and BRAF are common in low grade type I serous ovarian carcinomas [48, 49], which have higher mtDNA content than type II tumors that are frequently associated with p53 mutations. P53 is involved in the regulation of mitochondrial transcription and replication [50]. P53 also interacts with mitochondrial polymerase gamma to enhance mtDNA biogenesis [51]. Thus, depending on which nuclear genes are mutated, and at what stage, mtDNA content may vary. 

The relationship between mtDNA content and the expression of ER or PR has not been well characterized. Some reports showed no significant difference in mtDNA content between ER+/PR+ and ER−/PR− breast tumors [6, 33], however, the sample size was pretty small. With a large sample size in the present study, our results showed that ER+/PR+ tumors have a significantly higher mtDNA content compared to the tumors of ER−/PR−, ER+/PR−, or ER−/PR+. These results suggest that expression of ER and PR may stimulate mitochondrial biogenesis [52]. 

Somatic mtDNA mutations in D-loop region, where the origin of replication is located, may also alter the rate of mtDNA biosynthesis [32, 33, 45]. Our data also showed that tumors with somatic mutations in D-loop region have significantly decreased mtDNA content. In contrast, the majority of tumors harboring somatic alterations in the coding regions for the rRNA, tRNA, or mRNA have increased mtDNA content. Since alterations in the coding regions are more likely to result in functional change, the increase in mtDNA content may reflect a mechanism to compensate the reduction in mitochondrial function [43]. Studies of mtDNA content in HCC [32] and breast cancer [33] also demonstrated that mutations in D-loop does not correlate with mtDNA copy number in tumor [32, 33]. However, mutation analysis in these studies did not include the coding regions.

Advanced age, higher histological grade, larger tumor size, and later TNM stage are all prognostic factors for survival of cancer patients [17]. In this study, univariate and/or multivariate analysis revealed that advanced age, higher histological grade, larger tumor size, and later TNM stage all have a higher hazard of death than their lower counterparts. The results also showed that tumors with both very low and very high mtDNA content have higher hazard of death. Since the majority of the tumors with lower mtDNA content have larger tumor size, and high grade tumors have relatively higher mtDNA content, and invasive nature, these observations may simply reflect the link of hazard of death with the tumor size and tumor grade, respectively.

## 5. Conclusion

Our results indicate that the mtDNA content in tumor tissues changes with tumor size, grade, and ER/PR status. Significant deviation from the medium range is associated with poor survival. Low levels of mtDNA content suggest rapid tumor growth, while high levels of mtDNA content may indicate that tumors become invasive.

## Supplementary Material

The summary of somatic mtDNA alterations, mtDNA content, T/N ratio of mtDNA content, and corresponding clinico-pathological information of the subset of 74 pair of samples used for screening of somatic mutations.

## Figures and Tables

**Table 1:** Correlation of mtDNA content in breast cancer with clinicopathological features.

		mtDNA content in tumor tissue		T/N ratio of mtDNA content	
	*n*	Mean ± SD	*P* value*	Mean ± SD	*P *value*
Age (years)					

<40	56	592 ± 530		0.861 ± 0.561	
40–49	103	588 ± 473	0.603	0.967 ± 0.7	0.817
50–59	77	574 ± 515		0.956 ± 0.92	
≥60	57	577 ± 402		0.956 ± 0.849	

Tumor size (cm^3^)			(versus C)		(versus C)

A: ≤4.9	79	735 ± 412	4.06**e** − 07	1.088 ± 0.636	1.84**e** − 05
B: 5~14.9	66	574 ± 354	0.004	1.000 ± 0.556	0.002
C: 15–49.9	75	372 ± 248		0.696 ± 0.459	
D: ≥50	53	535 ± 385	0.306	0.818 ± 0.553	0.258

Grade					

0~I	18	669 ± 412		0.995 ± 0.681	
II	125	506 ± 318	**0.019 (versus 0~I**)	0.791 ± 0.531	0.827 (versus 0~I)
III	98	648 ± 432	0.383 (versus 0~I) **0.006 (versus II) **	1.128 ± 0.988	0.441 (versus 0~I) **0.033 (versus II) **

ER					

Negative	107	502 ± 405	*0.103*	0.810 ± 0.612	**0.047**
Positive	157	573 ± 374		1.057 ± 1.059	

PR					

Negative	100	494 ± 352	*0.061*	0.839 ± 0.633	*0.059*
Positive	154	597 ± 384		1.034 ± 0.772	

ER/PR					

A:ER,PR both − or either +	134	473 ± 316	**0.005**	0.837 ± 0.536	0.355
B: ER+/PR+	129	618 ± 381		0.930 ± 0.617	

Her2/neu receptor					

Negative	56	444 ± 333	*0.101*	0.727 ± 0.518	**0.023**
Positive	168	531 ± 346		1.127 ± 1.184	

*P values are from Mann-Whitney or Kruskal-Wallis test. The *P* values <0.05 are in bold, indicating significance; the *P* values >0.05, but <0.15 are in italic, indicating weak significance.

**Table 2:** Somatic alterations and alterations of mtDNA content.

	Regions with somatic alterations					Alteration of mtDNA content									
		mtDNA content in tumor tissue		T/N ratio		T/N ratio < 0.8					T/N ratio > 1.2				
	Mean ± SD	*P* value*	Mean ± SD	*P* value*	*n*	%	Odds	95% CI (Low-High)	*P* value **	*n*	%	Odds	95% CI (low-High)	*P* value **

A	D-loop only (*n* = 19)	496 ± 181	**<0.001** ^Δ^	0.65 ± 0.35	**<0.001** ^Δ^	11	57.9	1.38	0.46–4.14	0.591^Δ^	1	5.3	0.15	0.02–1.23	*0.081*
B	Coding with/ without D-loop (*n* = 15)	724 ± 369	**<0.001** ^Δ^ **<0.001** ^ΔΔ^	1.35 ± 0.71	**<0.001** ^Δ^ **<0.001** ^ΔΔ^	4	26.7	0.36 0.26	0.1–1.34 0.06–1.14	*0.141* ^Δ^ *0.091* ^ΔΔ^	9	60	3.95 27	1.14–13.73 2.81–26	**0.033** ^Δ^ **0.001** ^ΔΔ^
C	No (*n* = 40)	562 ± 310		1.22 ± 1.41		20	50				11	27.5			

T/N ratio: the ratio of mtDNA content in tumor tissue to the mtDNA content in surrounding normal tissue; SD: standard deviation; *: Mann-Whitney test; **: Fisher's exact test, ^Δ^versus no somatic alterations (C); ^ΔΔ^versus non-coding D-loop region alterations only (A). The *P* values <0.05 are in bold, indicating significance; the *P* values >0.05, but <0.15 are in italic, indicating weak significance.

**Table 3:** Univariate and multivariate COX regression survival analysis.

Variables	Group	*n*	Univariate		Multivariate	
			HR (95% CI)	*P* value	HR (95% CI)	*P* value
Grade	0–II	68	1.00		1.00	
	III	35	2.65 (1.22–5.74)	**0.014**	2.48 (1.12–5.51)	**0.025**
Size (CM^3^)	<15	62	1.00		1.00	
	≥15	73	3.01 (1.42–6.37)	**0.004**	2.51 (0.96–6.55)	*0.060*
Age (Years)	<50	78	1.00		1.00	
	≥50	70	1.76 (0.94–3.30)	*0.076*	3.75 (1.44–9.75)	**0.007**
mtDNA Content in Tumor	<400	50	2.17 (1.03–4.55)	**0.042**	3.39 (1.17–9.82)	**0.025**
	400–600	55	1.00		1.00	
	>600	43	1.37 (0.59–3.16)	0.463	4.05 (1.27–12.87)	**0.018**
Stage	0-I	14	1.00			
	II	62	1.67 (0.38–7.36)	0.397		
	III-IV	18	7.10 (1.57–32.18)	**0.011**		

The *P* values <0.05 are in bold, indicating significance; the *P* values >0.05, but <0.10 are in italic, indicating weak significance. HR: hazard ratio; 95% CI: 95% confidence interval.

## Abbreviations

mtDNA: Mitochondrial DNA
T/N ratio: The ratio of mtDNA content in tumor tissue to the mtDNA content in the surrounding normal tissue
ER: Estrogen receptor
PR: Progesterone receptor.
