# Supplementary material for: Mitochondrial DNA Content Varies with Pathological Characteristics of Breast Cancer
Source: J Oncol. 2011 Oct 17;2011:496189. doi: 10.1155/2011/496189 (PMC3199065; doi:10.1155/2011/496189)
Supplement: Supplementary file 1 — The summary of somatic mtDNA alterations, mtDNA content, T/N ratio of mtDNA content, and corresponding clinico-pathological information of the subset of 74 pair of samples used for screening of somatic mutations. [file 496189.f1.doc]

| **Table S1. Summary of the mtDNA alterations,mtDNA content and T/N ratio of mtDNA content** | | | | | | | | | | | | | | | |
| --- | --- | --- | --- | --- | --- | --- | --- | --- | --- | --- | --- | --- | --- | --- | --- |
| Sample# | Gene/ Region | Somatic alterations  (Normal to tumor) | Nucleotide in Mitomap | Normal to tumor pattern | Codon change | Amino acid change | Comments | mtDNA content | T/N ratio | age | Tumor size | Tumor grade | Stage | ER | PR |
| 102 | D-loop | C152T | T | hm->hm |  | - | Reported | 520 | 0.86 | 71 | 1.76 | 1 | na | na | na |
|  | D-loop | C204T | T | hm->hm |  | - | Reported |  |  |  |  |  |  |  |  |
|  | D-loop | A207G | G | hm->hm |  | - | Reported |  |  |  |  |  |  |  |  |
|  | D-loop | C16189T | T | hm->ht |  | - | Reported |  |  |  |  |  |  |  |  |
| 104 | D-loop | G73A | A | hm->hm |  | - | Reported | 459 | 0.66 | 48 | 1.1 | 2 |  | 3 | 2 |
|  | D-loop | C16325T | T | ht->hm |  | - | Reported |  |  |  |  |  |  |  |  |
|  | D-loop | T16519C | T | hm->hm |  | - | Reported |  |  |  |  |  |  |  |  |
| 106 | D-loop | C195T | T | hm->hm |  | - | Reported | 308 | 0.43 | 50 | 15.6 | 3 |  | 0 | 1 |
|  | D-loop | C16519T | T | hm->ht |  | - | Reported |  |  |  |  |  |  |  |  |
| 108 | D-loop | C16147T | C | hm->ht |  | - | Reported | 251 | 1.02 | 64 | 2.89 | 3 |  | 0 | 0 |
| 110 | D-loop | A16293G | A | hm->ht |  | - | Reported | 356 | 0.87 | 41 | 7.84 | 3 |  | 0 | 3 |
| 112 | ATP6 | T9131C | T | hm->hm | CTA>CCA | L202P | Novel; highly conserved | 118 | 0.07 | 68 | 27 | 3 |  | na | na |
| 114 | D-loop | T150C | C | hm->hm |  | - | Reported | 467 | 0.47 | 40 | 1.35 | 2 |  | na | na |
|  | D-loop | A185G | G | hm->hm |  | - | Reported |  |  |  |  |  |  |  |  |
|  | D-loop | G189A | A | hm->hm |  | - | Reported |  |  |  |  |  |  |  |  |
| 142 |  | (-) |  |  |  | - |  | 480 | 0.63 | 64 | 4.912 | 3 |  | 0 | 0 |
| 144 |  | (-) |  |  |  | - |  | 52 | 0.05 | 76 | 10.648 | 3 |  | 3 | 3 |
| 146 | D-loop | 303-309 ins CC (C9-C9/10/11) | C7 | hm->ht |  | - | Reported | 315 | 0.05 | 44 | 8 | 2 |  | 3 | 3 |
|  | D-loop | A16182C | A | hm->ht |  | - | Reported |  |  |  |  |  |  |  |  |
| 148 |  |  |  |  |  | - |  | 510 | 0.27 | 43 | na | 2 |  | 2 | 2 |
| 152 | D-loop | T16362C | T | ht->hm |  | - | Reported | 646 | 0.81 | 67 | 2.744 | na |  | 0 | 1 |
|  | D-loop | C16365T | C | ht->ht |  | - | Reported |  |  |  |  |  |  |  |  |
| 154 |  | (-) |  |  |  | - |  | 584 | 0.81 | 59 | 0.08 | 3 |  | 3 | 1 |
| 156 |  | (-) |  |  |  | - |  | 504 | 0.45 | 70 | na | na |  | na | na |
| 158 | D-loop | 303-309 ins C (C8-C8/9) | C7 | hm-> ht |  | - | Reported | 572 | 0.79 | 74 | 66.55 | 3 |  | 3 | 1 |
| 160 |  | (-) |  |  |  | - |  | 376 | 0.78 | na | na | na |  | na | na |
| 176 | D-loop | 303-309 ins C (C7/8-C8) | C7 | ht->hm |  | - | Reported | 508 | 0.89 | na | na | na |  | na | na |
|  | 16S rRNA | A1811G | A | hm->ht |  | - | Reported |  |  |  |  |  |  |  |  |
| 178 |  | (-) |  |  |  | - |  | 395 | 0.64 | na | na | na |  | na | na |
| 180 | D-loop | C16172T | T | ht->hm |  | - | Reported | 659 | 1.25 | na | na | na |  | na | na |
| 182 | ND2 | T4973C | T | ht->hm | GGT>GGC | M108M | Reported | 493 | 0.57 | na | na | na |  | na | na |
| 184 | D-loop | 303-309 del C (C8-C7) | C7 | hm->hm |  | - | Reported | 550 | 2.21 | na | na | na |  | na | na |
|  | ND2 | C4973T | T | hm->hm | GGC>GGT | M108M | Reported |  |  |  |  |  |  |  |  |
|  | ND2 | G5285A | A | hm->hm | AAG>AAA | K272K | Reported |  |  |  |  |  |  |  |  |
| 186 |  | (-) |  |  |  | - |  | 1404 | 2.49 | na | na | na |  | na | na |
| 201 |  | (-) |  |  |  | - |  | 574 | 0.57 | 65.2 | 58.8 | 2 |  | 0 | 0 |
| 204 |  | (-) |  |  |  | - |  | 444 | 0.62 | 46.6 | 3.9 | 3 |  | 1 | 3 |
| 207 |  | (-) |  |  |  | - |  | 542 | 0.52 | 49.5 | 0.75 | 1 |  | 1 | 2 |
| 210 |  | (-) |  |  |  | - |  | 345 | 0.62 | 44.6 | 9 | 3 |  | 2 | 3 |
| 212 |  | (-) |  |  |  | - |  | 1168 | 2.05 | 40.2 | 1.5 | 3 |  | 2 | 2 |
| 214 |  | (-) |  |  |  | - |  | 612 | 0.51 | 35.7 | 2431 | 2 |  | 0 | 0 |
| 217 | D-loop | 303-309 del C (C8/7-C7/8) | C7 | ht->ht |  | - | Reported | 939 | 1.11 | 29.9 | 14.49 | 2 |  | 3 | 3 |
| 219 | ATP6 | C9176T | T | ht->ht | CCA>CTA | P217L | Confirmed pathogenic mutation | 623 | 1.34 | 47.6 | 21.875 | 2 |  | 0 | 0 |
| 222 |  | (-) |  |  |  | - |  | 662 | 0.7 | 40.4 | 25.84 | 2 |  | 0 | 0 |
| 225 | D-loop | 303-309 ins C (C8/9-C9/8) | C7 | ht->ht |  | - | Reported | 1351 | 1.3 | 34.9 | 3.375 | 2 |  | 2 | 3 |
|  | tRNA Leu2 | C12284T |  | hm->ht |  | - | Reported |  |  |  |  |  |  |  |  |
| 227 | D-loop | T514C | C | ht->hm |  | - | Novel | 408 | 0.29 | 44.9 | 3.35 | 2 |  | 2 | 3 |
| 230 |  | (-) |  |  |  | - |  | 364 | 0.48 | 73.6 | 16.5 | 2 |  | 1 | 1 |
| 233 | COI | C6680T | T | hm->hm | ACC>ACT | T259T | Reported | 1435 | 2.63 | 46.6 | 14 | 1 |  | 2 | 3 |
|  | COIII, | C9540T | T | ht->ht | CTA>TTA | L112L | Reported |  |  |  |  |  |  |  |  |
| 236 | D-loop | 303-309 ins C (C7/8-C8) | C7 | ht->hm |  | - | Reported | 461 | 0.52 | 52 | 36 | 3 |  | 3 | 1 |
| 238 | D-loop | G73A | A | ht->hm |  | - | Reported | 1266 | 2.21 | 53.3 | 1.44 | 3 |  | 0 | 0 |
|  | COII | C8110T | T | hm->hm | ATC>ATT | I 175I | Reported |  |  |  |  |  |  |  |  |
|  | D-loop | C150T | C | ht->hm |  | - | Reported |  |  |  |  |  |  |  |  |
|  | D-loop | C199T | T | ht->hm |  | - | Reported |  |  |  |  |  |  |  |  |
|  | D-loop | 303-309 ins C (C7-C8) | C7 | ht->hm |  | - | Reported |  |  |  |  |  |  |  |  |
|  | 12s rRNA | C752T | C | hm->hm |  |  | Reported |  |  |  |  |  |  |  |  |
|  | ATP6 | A9180G | A | hm->ht | GTA>GTG | V218V | Reported |  |  |  |  |  |  |  |  |
|  | 12S rRNA | G1438A | A | hm->hm |  | - | Reported |  |  |  |  |  |  |  |  |
|  | COIII | C9540T | T | hm->hm | CTA>TTA | L112L | Reported |  |  |  |  |  |  |  |  |
|  | D-loop | 514-523 del CA(CA5-CA4) | CA5 | hm-> hm |  | - | Reported |  |  |  |  |  |  |  |  |
|  | COIII | T9698C | T | hm->ht | CTT>CTC | L164L | Reported |  |  |  |  |  |  |  |  |
|  | ND5 | T12405C | C | hm->hm | CTC>CTT | L23L | Reported |  |  |  |  |  |  |  |  |
|  | ND5 | C12811T | T | hm->hm | CAC>TAC | H159Y | Reported ; not conserved |  |  |  |  |  |  |  |  |
| 241 | D-loop | 303-309 ins C (C8-C9) | C7 | ht->hm |  | - | Reported | 824 | 1.51 | 58.4 | 2.783 | 2 |  | 2 | 2 |
|  | 16s rRNA | G3145A |  | ht->hm |  | - | Reported |  |  |  |  |  |  |  |  |
| 244 |  | (-) |  |  |  | - |  | 1266 | 1.33 | 85 | 22.5 | 2 |  | 2 | 1 |
| 246 |  | (-) |  |  |  | - |  | 746 | 1.16 | 46.6 | 6.44 | 2 |  | 1 | 2 |
| 249 | D-loop | 303-309 ins C (C8/9-C9/10) | C7 | ht->ht |  | - | Reported | 610 | 0.53 | 66.8 | 0.48 | 1 |  | 1 | 1 |
| 252 |  | (-) |  |  |  | - |  | 75 | 1.85 | 45.6 | 0.002 | 2 |  | 0 | 0 |
| 255 | tRNA-Ser2 | G12293A | G | hm->ht |  | - | Reported | 869 | 1.54 | 75.2 | 7 | 3 |  | 0 | 0 |
| 258 | D-loop | 303-309 ins C (C8/C9-C9) | C7 | ht-> ht |  | - | Reported | 518 | 0.67 | 64.6 | 2.16 | 2 |  | 2 | 1 |
|  | ND2 | C4646T | T | ht->hm | TAC>TAT | Y59Y | Reported |  |  |  |  |  |  |  |  |
| 261 | D-loop | 303-309 del C (C8-C7/C8) | C7 | hm->ht |  | - | Reported | 800 | 0.2 | 85.2 | 72 | 2 |  | 3 | 1 |
| 264 |  | (-) |  |  |  | - |  | 442 | 1.06 | 35.8 | 0.96 | 2 |  | 1 | 1 |
| 267 | D-loop | 303-309 del C (C8-C7) | C7 | hm->hm |  | - | Reported | 631 | 0.41 | 62.7 | 8.28 | 3 |  | 0 | 0 |
|  | D-loop | C311T | C | hm->ht |  | - | Reported |  |  |  |  |  |  |  |  |
| 270 |  | (-) |  |  |  | - |  | 369 | 0.52 | 33.2 | 29.7 | 2 |  | 2 | 3 |
| 273 |  | (-) |  |  |  | - |  | 86 | 0.39 | 52.8 | 35 | 2 |  | 1 | 1 |
| 296 |  | (-) |  |  |  | - |  | 511 | 0.93 | 39 | 11.2 | 2 |  | 1 | 1 |
| 299 |  | (-) |  |  |  | - |  | 647 | 1.58 | 63.8 | 2464 | 3 |  | 0 | 0 |
| 302 | D-loop | 303-309 del C (C8/9->C7/8) | C7 | ht->ht |  | - | Reported | 529 | 1.2 | 51 | 1.95 | 3 |  | 2 | 0 |
| 305 |  | (-) |  |  |  | - |  | 473 | 0.65 | 57.1 | 8.1 | 3 |  | 1 | 2 |
| 308 |  | (-) |  |  |  | - |  | 578 | 1.45 | 47.7 | 15 | 3 |  | 2 | 0 |
| 311 |  | (-) |  |  |  | - |  | 1178 | 2.08 | 54 | 301 | 2 |  | 2 | 2 |
| 314 | D-loop | C16185T | C | ht->ht |  | - | Reported | 738 | 0.67 | 38.8 | 38.41 | 2 |  | 2 | 2 |
|  | COIII | T9540C | T | ht->hm | TTA>CTA | L112L | Reported |  |  |  |  |  |  |  |  |
| 317 |  | (-) |  |  |  | - |  | 392 | 0.62 | 37.1 | 19.25 | 2 |  | 1 | 2 |
| 320 |  | (-) |  |  |  | - |  | 596 | 0.76 | 42.1 | 20.25 | 3 |  | 1 | 1 |
| 323 | D-loop | A178G | A | hm->ht |  | - | Reported | 259 | 0.85 | 42.5 | 1.3 | 2 |  | 1 | 3 |
| 326 |  | (-) |  |  |  | - |  | 407 | 0.63 | 34 | 176.472 | 3 |  | 1 | 1 |
| 328 |  | (-) |  |  |  | - |  | 46 | 0.69 | 39.1 | 286 | 2 |  | 0 | 0 |
| 331 |  | (-) |  |  |  | - |  | 456 | 0.92 | 56.2 | 1.472 | 3 |  | 1 | 1 |
| 334 | 16s rRNA | G1858A | G | hm->ht |  | - | Reported | 575 | 1.64 | 52.4 | 11.25 | 3 |  | 1 | 2 |
| 337 |  | (-) |  |  |  | - |  | 90 | 1.2 | 38.3 | 7.5 | 2 |  | na | na |
| 340 |  | (-) |  |  |  | - |  | 642 | 0.94 | 39.1 | 2.88 | na |  | 2 | 2 |
| 342 |  | (-) |  |  |  | - |  | 764 | 0.83 | 33.4 | 20.81 | 3 |  | 0 | 0 |
| 345 | COII | C7856A | A | ht->ht | CAC>AAC | H91N | Novel; Relatively conserved | 571 | 1.04 | 66 | 6.75 | 2 |  | 3 | 2 |
| 348 |  | (-) |  |  |  | - |  | 838 | 1.79 | 52.1 | 6.75 | 2 |  | 0 | 0 |
| 351 | D-loop | C16092T | T | ht->hm |  | - | Reported | 351 | 0.65 | 51.1 | 17.112 | 2 |  | 3 | 1 |
| 354 |  | (-) |  |  |  | - |  | 704 | 8.86 | 37.2 | 0.384 | 3 |  | 3 | 3 |
| 357 |  | (-) |  |  |  | - |  | 712 | 1.21 | 53.7 | 28.86 | 2 |  | 2 | 3 |
| 359 |  | (-) |  |  |  | - |  | 510 | 0.89 | 69.5 | 117 | 3 |  | 0 | 0 |
| 362 |  | (-) |  |  |  | - |  | 828 | 3.54 | 54 | 121.5 | 3 |  | 0 | 0 |
| 365 | 16S rRNA | A3151G | A | hm->ht |  | - | Novel | 430 | 1.96 | 69.1 | 1.98 | 2 |  | 3 | 3 |
| T/N: T/N ratio of mtDNA content; hm: homoplasmy; ht: heteroplasmy; (-): no somatic mtDNA alterations; na: not available | | | | | | | | | | | | | | | |
